# Ingested Nasopharyngeal Swab for Viral Testing Retained in a Child’s Duodenum

**DOI:** 10.1097/PG9.0000000000000295

**Published:** 2023-02-28

**Authors:** Ayako Miyazawa, Ryusuke Nambu, Masashi Yoshida, Takahiro Hosokawa, Itaru Iwama

**Affiliations:** From the * Division of Gastroenterology and Hepatology, Saitama Children’s Medical Center, Saitama, Japan; † Department of Radiology, Saitama Children’s Medical Center, Saitama, Japan.

We face a variety of foreign object ingestions. However, we agree that any object longer than 6 cm is not likely to be excreted spontaneously (1). This is the case of a child ingesting a nasal swab accidentally that stayed in the duodenum.

The nasal test for coronavirus disease 2019 (COVID-19) using a flock swab with breakpoints was administered to a 4-year-old boy presenting with fever at a clinic. Although his mother held him, he moved during the test, thus breaking the swab. On computed tomography, the ingested swab was in the stomach and advanced to the third portion of the duodenum after 5 days (Fig. 1A); spontaneous passage was expected. Four weeks since the entry, he was referred to our hospital. No gastrointestinal symptoms or food intake issues were noted. On endoscopy, the foreign body remained in the same location (Fig. 1B), with a mildly red mucosa but without ulceration. The 9-cm-long swab was removed (Fig. 2), and he was discharged the next day without complications.

**FIGURE 1.**
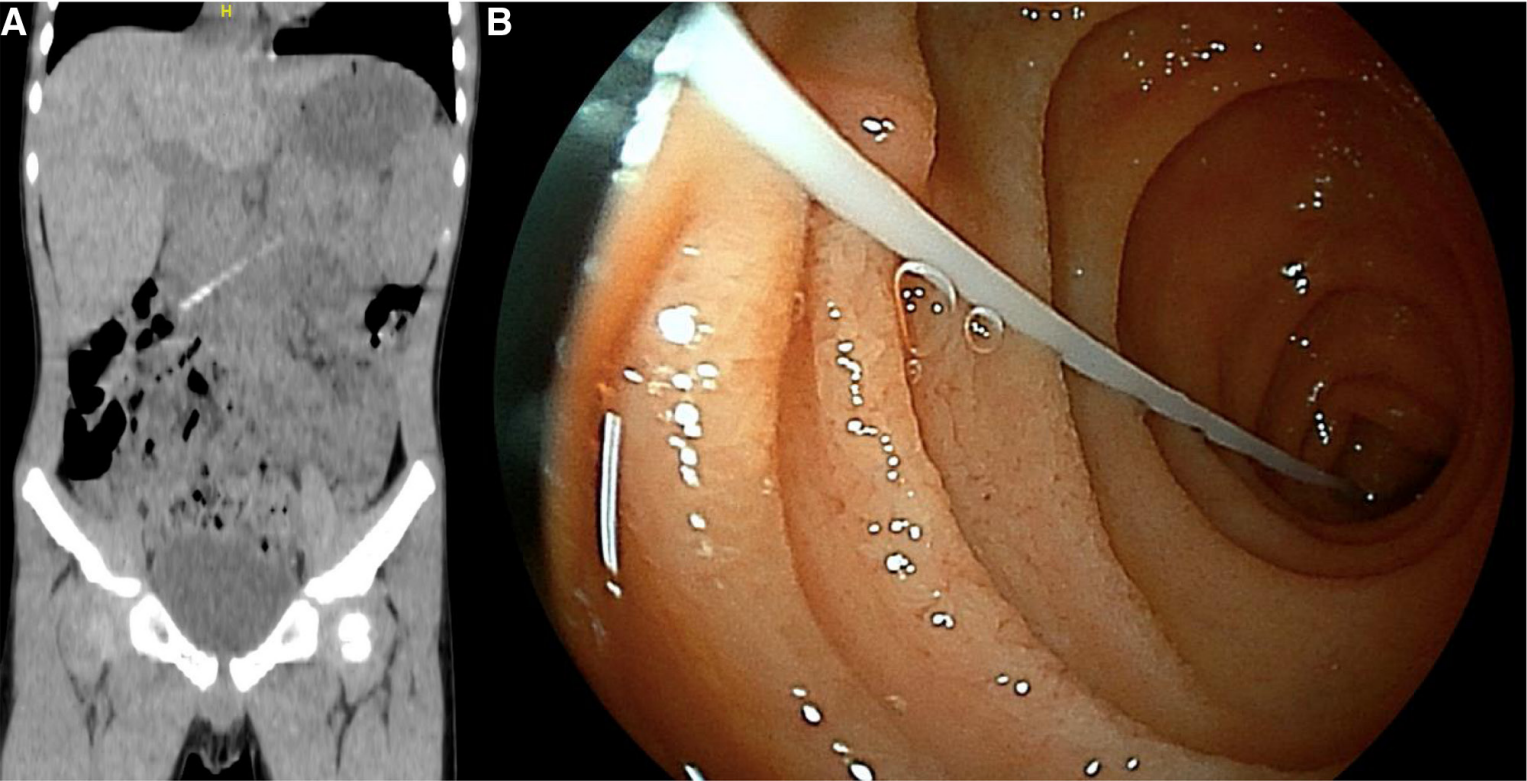
Nasal swab retained in the third portion of the duodenum. A) Computed tomography 5 d after the accidental ingestion. B) Endoscopic image. The top of the nasal swab was on the anorectal side. Only mild redness was seen on the contact surface.

**FIGURE 2.**
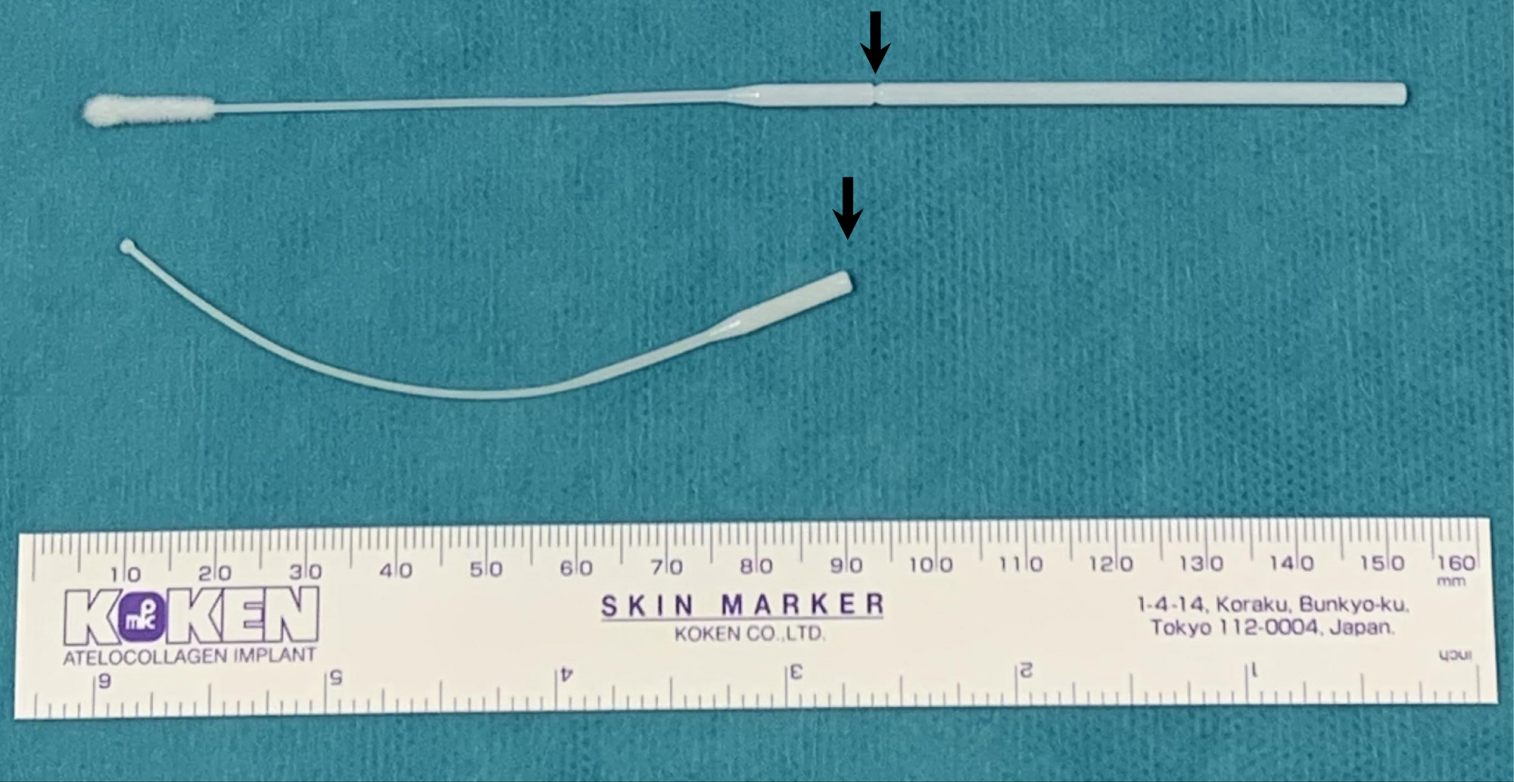
Top: Unused swab. Bottom: Swab removed from the patient, fractured at the breakpoint (arrow).

Adverse events of nasal swab testing, including accidental ingestion even in an infant, have become common due to the increased number of COVID-19–related tests with the widespread use of nasal swabs with a breakpoint at its handle (2–4). In this case, spontaneous excretion was expected due to its flexibility. However, the length of the swab made it difficult. Nasal swabs can even perforate the sigmoid colon of adults (5). If nasal swab with a breakpoint is ingested, endoscopic removal should be attempted.

## ACKNOWLEDGMENTS

We would like to express our sincere appreciation to the parents of the child who gave us their consent of this case report.
